# A ROD9 island encoded gene in Salmonella Enteritidis plays an important role in acid tolerance response and helps in systemic infection in mice

**DOI:** 10.1080/21505594.2020.1733203

**Published:** 2020-03-01

**Authors:** Susmita Das, Shilpa Ray, Aryashree Arunima, Bikash Sahu, Mrutyunjay Suar

**Affiliations:** School of Biotechnology, KIIT University, Bhubaneswar, India

**Keywords:** *Salmonella* Enteritidis, ROD9, *SEN1008*, acid tolerance, C57Bl/6, inflammation

## Abstract

*Salmonella*, like other pathogenic bacteria has undergone multiple genomic alterations to adapt itself into specific host environments executing varied degrees of virulence through evolution. Such variations in genome content have been assumed to lead the closely related non-typhoidal serovars, *S*. Enteritidis, and *S*. Typhimurium to exhibit Type Three Secretion System −2 (T3SS-2) based diverse colonization and inflammation kinetics. Mutually exclusive genes present in either of the serovars are recently being studied and in our currentwork, we focused on a particular island ROD9, present in *S*. Enteritidis but not in *S*. Typhimurium. Earlier reports have identified a few genes from this island to be responsible for virulence *in vitro* as well as *in vivo*. In this study, we have identified another gene, *SEN1008* from the same island encoding a hypothetical protein to be a potential virulence determinant showing systemic attenuation upon mutation in C57BL/6 mice infection model. The isogenic mutant strain displayed reduced adhesion to epithelial cells *in vitro* as well as was highly immotile. It was also deficient in intracellular replication *in vitro*, with a highly suppressed SPI-2and failed to cause acute colitis at 72-h p.i.*in vivo*. Moreover, on acid exposure, *SEN1008* showed 17 folds and 2 fold up-regulations during adaptation and challenge phases,respectively and Δ*SEN1008* failed to survive during ATR assay, indicating its role under acid stress. Together, our findings suggested Δ*SEN1008* to be significantly attenuated and we propose this gene to be a potent factor responsible for *S*. Enteritidis pathogenesis.

## Introduction

*Salmonella enterica* is Gram-negative motile bacteria capable of causing non-typhoidal food borne illnesses and even systemic infections in a wide variety of hosts, including humans[1]. Serovars *S*. Enteritidis and *S*. Typhimurium are two major pathogens causing salmonellosis in more than 1 million population in U.S. per year [2]. Its mode of infection is basically characterized by its two main pathogenicity islands, SPI-1, and SPI-2 encoding the complex Type III secretion systems, TTSS-1, and TTSS-2, respectively, [3]. Post entry into the host gut, *Salmonella* encounters the intestinal epithelial cells and immediately initiates adhesion with the help of a number of fimbrial or non-fimbrial adhesins [4]. Following adhesion, the bacteria recruits its TTSS-1 machinery and injects effectors into the host cytosol leading to its engulfment via a complex set of proteins ensuring bacterial colonization. This invasion process also triggers early inflammation in the host, as soon as 8–24 hours p.i. through activation of pro-inflammatory cytokine pathways [5]. Inside the host, *Salmonella* forms a comfortable niche named *Salmonella*-containing vacuole (SCV) to replicate and protect itself from the harsh immune responses of the host [6]. The process of formation of SCV and intravacuolar bacterial replication inside the phagocytic as well as non-phagocytic cells are driven by the effectors of TTSS-2, encoded by genes present in SPI-2 [7]. Now, SPI-2 is also responsible for the alternative pathway of infection that allows the bacteria to be sampled by DCs from the lamina propria, following their transfer to macrophages which next transport them to mesenteric lymph nodes (mLN) [8]. Further, the bacteria are disseminated frommLN to the systemic organs such as liver and spleen through the blood stream and lymphatic system [9]. Thus,SPI-2 strongly contributes to systemic infection as well as late inflammation that occur after 48–72 h of infection through MyD88 pathway [8,10].

During its course of infection, *Salmonella* is subjected to a range of hostile environments inside the host, including stomach acid, ROS, bile salts, osmolarity shocks, etc. [11]. One of the major stress factors to overcome for the bacteria is low gastric pH and the mechanism to sense and adapt to it is critical for its survival. The bacteria is exposed to acidic pH primarily, in the stomach and macrophages and this strategy helps in killing the pathogen as well as enhancing other host defense pathways such as phagolysosomal integration, production of acid hydrolases, etc. [12,13]. Following acid stress, *Salmonella* undergoes an adaptation phase where it adjusts itself to a sub-lethal pH of 4.4–5.8 for a generation to gain the power to survive at such acidic conditions and to further fight severe acid exposures (pH 3) through the expression of a number of ASPs [14]. The phenomenon of adaptation to acid stress is termed as acid tolerance response and has been reported to play an important role in bacterial survival and infection in mice models [15].

Our previous study showed that, in ROD9 (or as it is referred, SPI-19) island of *S*. Enteritidis, a gene *SEN1005* encoding a hypothetical protein contributed to the infection profile by altering SPI-1 and other virulence-related genes expression [16]. In the process to investigate other virulent genes from the same island, we identified another gene *SEN1008* encoding a novel protein with a Sel1-like repeat domain. The isogenic mutant strain already showed adhesion deficiency in epithelial cells and reduced uptake by macrophages *in vitro. In silico* studies via pBLAST against the Virulence Database revealed the protein to consist similar motifs with that of other virulent proteins, viz., enhanced entry protein (EnhC), fimbrial biogenesis, and twitching motility protein (PilF), type IV secretion Dot/Icm protein in *Legionella pneumophila*, a Gram-negative pathogen causing gastroenteritis. The present work carried out with the isogenic mutant strain, Δ*SEN1008* showed involvement of the gene in acid tolerance response as well as in bacterial motility. Moreover, the mutant strain displayed a drastic reduction in bacterial replication in macrophages and failed to show significant histopathological signs at day 3 p.i.Δ*SEN1008*was systemically attenuated that corresponded to low expression levels of SPI-2 effectors in infected mice tissues as well as in *in vitro* cultures. All these observations led us to assume *SEN1008* to be involved in bacterial acid tolerance adaptation mechanism and to play a pivotal role in systemic spread of the pathogen.

## Materials and methods

### Bacterial strains and plasmids used in this study and growth parameters

The bacterial strains along with the plasmids used in the present study are listed in Table 1. All the bacterial strains were grown in Luria-Bertani (LB) medium with required antibiotics at appropriate concentrations such as streptomycin 50 µg/ml, kanamycin 50 µg/ml, ampicillin 100 µg/ml, chloramphenicol 20 µg/ml. To set up SPI-1 inducing growth conditions, LB broth was prepared with a higher NaCl concentration of 0.3 M and the cultures were subjected to low aeration (120 rpm) at 37°C for 12 h. For *in vitro* and *in vivo* infection experiments, overnight grown strains were further sub-cultured into fresh SPI-1 inducing LB broth to reach an O.D_600_of 0.6.

**Table 1.** Bacterial strains and plasmids.

Strains	Description	References
WT	*Salmonella enterica* serovar EnteritidisP125109	[26]
∆*SEN1008*	*SEN1008*::Km (Mutant strain)	[16]
Δ*SEN1008*/pCH112-1008	pCH112-1008 in *SEN1008*::Km (Complemented strain)	This study
*∆invC*	SEN*invC*::aphT (Mutant strain)	[26]
*∆ssaV*	*S*.EnteritidisΔ*ssaV*; Sm^r^	This study
**Plasmids**		
pKD4	Template plasmid; FRT-*aphT*-FRT (containing kanamycin resistance gene) bla FRT aph FRT PS1 PS2 oriR6 K	[9]
pKD46	Red recombinase expression plasmid *bla*p*BAD gam bet exo*pSC101 *ori*TS (containing ampicillin resistance gene)	[9]
pCP20	FLP recombinase expression plasmid	[9]
pCH112	hilA ORF cloned into pBAD/myc-His; *ori*pBR322	[19]
pCH112-1008	*SEN1008* expressing vector; *SEN1008* cloned with its 1000bp upstream region by replacing HilA in *ori*pBR322 of pCH112	This study
pCJLA-GFP	GFP-plasmid used to tag strains	[22]

Bacterial growth rate in LB media was determined for the three strains *viz*. WT, Δ*SEN1008* and Δ*SEN1008/*pCH112-1008to compare the growth kinetics over a span of 8 h at 37°C at 150 rpm. Hence, bacterial cultures were harvested at regular time intervals, followed by serial dilutions and plating on LB agar forcfu counts.

Bacterial cell propagation in MEM was done according to literature [17]. Briefly, it was obtained by culturing bacterial strains in triplicate over a period of 7 h at 37ºC, 180rpm. For the initial 1-h post subculture, the plating of appropriate dilutions was done every 30 min, after which plating was performed at each hour interval to determine cfu counts.

### Preparation of deletion mutants and plasmid-based complemented strain

For this study, we used the deletion mutant created in our previous work [16]using the Lambda Red recombination system where the desired gene was replaced by a kanamycin resistance cassette using template plasmid pKD4 and helper plasmid pKD46 [18]. Primers used are listed in Table S3.1.

The complemented strain for Δ*SEN1008*was done using the plasmid pCH112 [19]. Briefly, the *SEN1008*gene, along with its 1000 bp upstream nucleotide sequence was cloned in between NcoI and XbaI sites in pCH112, followed by clone confirmation by PCR with insert specific primers and restriction digestion to allow insert release. Next, the transformation of the clone was performed in the *ΔSEN1008* strain.

### Mammalian cell adhesion assay

Cell adhesion assay was performed as earlier [20]. In short, the HCT116 cell line was maintained in Dulbecco’s modified Eagle’s medium (DMEM) (Gibco), supplemented with 10% Fetal Bovine Serum (FBS). Cells were seeded in 24 well plates and incubated for a day to reach 80% confluency. Under SPI-1 inducing conditions, sub-cultured bacterial strains were taken at aroundO. D_600_ of 0.6. They were then diluted at 1:5 ratio in LB. Cell lines were thrice washed with PBS, supplemented with DMEM (without antibiotics) and both the bacterial inoculums and mammalian cells were incubated on ice for 15 min prior to infection. Cells were then infected at an MOI of 10 bacteria/cell and kept for next half an hour on ice to restrict invasion, immediately followed by lysis with 0.1% sodium deoxycholate, serially diluted and plated to determine the number of adhering bacteria. *invC*, being a SPI-1 encoded T3SS-1 apparatus gene responsible for bacterial invasion, its isogenic mutant strain was used a positive control in this study. Theassaywasperformed thrice in triplicates. The result was calculated as (cfu obtained after infection assay)/(PID or Pre-inoculum density). The value for WT strain was considered to be 100% and the rest were estimated accordingly.

### Bacterial uptake and intracellular replication assay in macrophages

The assays were performed as explained previously [21]. In brief, the murine macrophage cell line RAW264.7, cultured in Dulbecco’s modified Eagle’s medium (DMEM) (Gibco), supplemented with 10% FBS were seeded in 24 well plates and kept for a day before infection. Overnight grown bacterial strains were resuspended in PBS and diluted to 10^8^cells/ml in DMEM (free of antibiotics). Macrophage cell lines were then infected with a MOI of 10 bacteria/cell, followed by incubation for 50 min in 5% CO_2_incubator. The media was then exchanged with DMEM (Gibco), supplemented with gentamicin to kill the extracellular bacteria. In order to study uptake, at 2-h p.i., cells were lysed with 0.1% Triton X-100 (HIMedia), serially diluted and plated to determine the number of bacteria engulfed by the macrophages. The result was evaluated as (cfu obtained 2 h p.i.)/(PID or Pre-inoculum density). The value for WT strain was considered to be 100% and the rest were estimated accordingly. For intracellular replication assay, 2-h post-antibiotic treatment, the media were changed with fresh DMEM containing a lower concentration of gentamicin and left for 24 h at 37°C to allow the bacteria to replicate themselves inside the macrophages. Following day, the cells were washed, lysed with Triton X-100 (HIMedia), serially diluted, and plated to enumerate viable bacterial counts. The fold replication was obtained as the cfu at 24-h p.i. as compared to that at 2 h p.i. for each strain. The experiment was repeated thrice.

### Fluorescence microscopy

Fluorescence microscopy was used to study adhesion of *Salmonella*,as described earlier [22] with WT and mutant *S*. Enteritidis, each transformed with the constitutively expressing GFP plasmid pCJLA. A MOI of 20 bacteria/cell was used in this case. Precisely, the HCT116 cells were seeded on coverslips immersed in DMEM (Gibco) at a density of 5 × 10^4^ cells/well and incubated overnight at 37°C with 5% CO_2_. Next day, the experiment was carried out on ice following the usual adhesion protocol. After infection, the coverslips were taken out, washed with chilled PBS, fixed with 4% paraformaldehyde (PFA) (Sigma-Aldrich), blocked with 5% BSA, and bacteria were then visualized by AMG EVOS fl LED Fluorescent microscope under 10X objective.

### ATR assay

Log-phase ATR assay was induced as mentioned in the literature [17]. Briefly, overnight grown WT, Δ*SEN1008* and Δ*SEN1008/*pCH112-1008cultures in 5 ml MEM (37ºC, 180rpm) were sub-cultured (1:100 dilution) till an OD_600_ of 0.4 were reached. Next, cultures to be adapted were given a pH 4.4 (±0.1) with 3 N HCl being added to the media and incubated for an hour. Un-adapted cultures were left aside to grow till the challenge phase. Post adaptation phase, cultures were typically challenged at pH 3.1 (±0.1) in a similar fashion and incubated further for 1, 2 and 4 h. Colony forming units were determined using serial dilutions and plating on agar medium. Survival percentage was calculated by dividing the CFU at indicated time points p.c. by CFU prior to challenge and multiplying it by 100. Experiment was performed thrice.

### Animal ethical statement

The *invivo*mice infection study was granted by the Institutional Animal Ethics Committee (IAEC) and all the experiments were done maintaining the strict rules and regulations set by the board ensuring minimum distresstothemice involved. The approval number for the same is KSBT/IAEC/2017/MEET-2/A4.

### Mouse infection study and histopathological evaluation

C57BL/6 mice were housed in the animal house facility of School of Biotechnology, KIIT University, Bhubaneswar, Odisha, India for a week to adjust with the experimental environment. The bacterial strains WT, Δ*SEN1008*, Δ*SEN1008/*pCH112-1008 were grown in SPI-1 inducing LB media for 12 h, followed by a subculture to obtain ~10^7^cfu. For this study, streptomycin-pretreated mouse model was used as reported earlier [23]. Four groups (n = 5) were fed orally with individual strains mentioned above and PBS (as negative control) with the help of sterilegavages. Fecal sheddingwas collected from each mouse at day 1 and day 2p.i. to confirm bacterial colonization in the gut. At 3 days p.i., all the mice were euthanized to determine the bacterial load in the mesenteric lymph node (mLN), spleen, liver, and cecal content. This was achieved after homogenizing the tissues in PBS, serially diluting them and plating on Mac Conkey agar plates, supplemented with streptomycin. Apart from this, cecal tissues were cryopreserved in cryomatrixand 5 µm sections were obtained using cryotome, followed by HE-staining and the histopathological score was estimated by two blinded pathologists on the basis of parameters such as formation of submucosal edema, absence of goblet cells, infiltration of polymorphonuclear neutrophils (PMNs) and epithelial cell disintegrity [24]. The path scores were given based on the mentioned parameters out of a total 13 arbitrary unit (a.u) corresponding to varied degrees of inflammation: healthy intestines without inflammation (0); negligible or very little inflammation (1–2); slightly inflamed (3–4); moderately inflammed (5–8); and acute inflammation (9–13).

### Creation of tagged strains of WT and ΔSEN1008 S. Enteritidis, competitive invivo infection study and qPCR

To create WITS, *S*. Enteritidis WT was tagged with specific barcode sequences (nucleotides) as explained earlier [25]. Before tagging, the kanamycin resistance cassette was removed from the mutant strain using pCP20 flip-recombinase plasmid to generate a clean mutant with streptomycin resistant. WITS-2 was tagged to the WT strain whereas WITS-11 and WITS-21 were tagged individually to the mutant strain by conventional phage transduction as described previously [26,27]. *ΔSEN1008* tagged WITS-11 strain was further transformed with pCH112-1008 plasmid to prepare the complemented tagged strain. The WITS tagged colonies were selected on LB agar plates supplemented with 50 µg/ml kanamycin and confirmed with PCR using primers specific to unique tags and a common primer to *ydgA*, located near the integration site. An additional PCR with *SEN1008* specific primers were run to confirm the deletion of the gene in the mutant strain after being tagged. Competitive mouse infection assay was performed using the protocol mentioned in the literature [27]. In brief, the tagged strains were grown individually in LB overnight, followed by subculture till mid-log phase and orally fed to 5 mice in 1:1:1 ratio, the total inoculum pool being ~10^7^cfu. At day 3p.i., the mice were euthanizedandmLN, spleen, liver, and cecal content were collected to isolate genomic DNA using DNeasyBlood& Tissue Kits (Qiagen), followed by qRT-PCR assay with WITS specific primers. The relative abundance of different strains in each organ was calculated in terms of ratio of WITS-11 and WITS-21 with respect to WITS-2 (i.e. WT). All the primers used in this experiment are listed in Table S1.

### Motility assay

Bacterial motility was evaluated on 0.3% w/v soft LB agar plates. Overnight grown bacteria cultures were all adjusted to same O.D and drop inoculation was done at the center of the plate as done previously (16). Five hours post incubation at 37°C, the motility of the bacteria was assessed by measuring the diameter of the bacterial colony spread around the drop. The experiment was performed thrice in triplicates.

### Flow cytometry

Bacterial uptake and replication assays in macrophage cell line RAW264.7 were performed as usual, but with GFP-tagged WT and mutant strain (Δ*SEN1008*), after the transformation of each with plasmid pCJLA-GFP [22]. The macrophages that phagocytosed the bacteria and survived till 24 h expressed GFP, whose intensity was measured by FACScanto^TM^ II flow cytometer.

### RNA extraction and real-time PCR assays

For RNA isolation from infected mice organs, mLN and spleen tissues were collected and snap-frozen at once in liquid nitrogen, followed by mechanical grinding in an RNase-free mortar pestle. RNA was extracted using Trizol reagent (Ambion), followed by RNase-free DNaseI treatment (Thermo Fisher Scientific) and cDNA synthesis using kit from HIMedia. With the diluted and normalized cDNA templates, quantitative RT PCR was performed using Kapa Sybr Fast qPCR Master Mix (2x; Kapa Biosystems, USA). 16 s rRNA gene was used as housekeeping gene. The qRT-PCR data were presented corresponding to the fold-change differences in gene expression in *ΔSEN1008* with respect to WT.

For the ATR assay, *S*. Enteritidis RNA was extracted from bacterial cultures growing at neutral pH (7.6), 1-h post-adaptation pH (4.4) and 1 hour post-challenge pH (3.1), followed by subsequent purification, cDNA synthesis and qRT-PCR analysis as already discussed. Guanylate monophosphate kinase (*gmK)* gene was used as housekeeping gene during ATR assay [17]. For fimbrial gene expression, Δ*SEN1008* and WT were grown in Luria-Bertani (LB) broth, sub-cultured for 4 h (37°C, 120 rpm), whereas overnight grown cultures were used to check*in vitro* SPI-2 genes and flagellar assembly genes expression and followed by usual RNA extraction, DNaseI purification, cDNA synthesis, and qRT-PCR study as mentioned. 16 s rRNA gene was used as housekeeping gene. Experiments were repeated thrice in triplicates. All the primers used are listed in supplementary Table S1.

### Cytokine analysis from serum samples by western blotting

For cytokine analysis, blood samples were drawn from infected mice at 72 h p.i.and stored after centrifuging them at 100xg for 10 min at 4°C [9]. For western blot [27], the collected serum samples were quantified using Bradford method of protein estimation and subsequently run on SDSpolyacrylamidegels and electro-transferred to PVDF membrane using a semi-dry blot. The membrane was then treated with 5% BSA in TBST (20 mM Tris HCl [pH 7.6], 0.15 M sodium chloride,and 0.5% Tween 20), for 2 h at RT and further incubated with respective 1° antibodies (Cell Signaling, USA) in 1% BSA in TBST (1:2000 dilution) for overnight on a rocker at 4°C. After washing thrice with TBST, the membrane was further treated with horseradish peroxidase conjugated appropriate 2° antibodies (anti-mouse or anti-rabbit Ig) for 1.5 h. Following three TBST washes, the blots were developed with the enhanced chemo-luminescence kit (Abcam, UK) as mentioned in the manual.The study was repeated thrice. The blots were quantified using ImageJ software [28].

### Statistical analysis

All the assays were performed minimum thrice in triplicates and graphs were plotted showing mean and standard deviation. The data were statistically verified with t-tests and ANOVA with the help of GraphPad Prism version 6.0.

## Results

### ΔSEN1008 displays reduced adhesion in vitro

**Figure 1.**
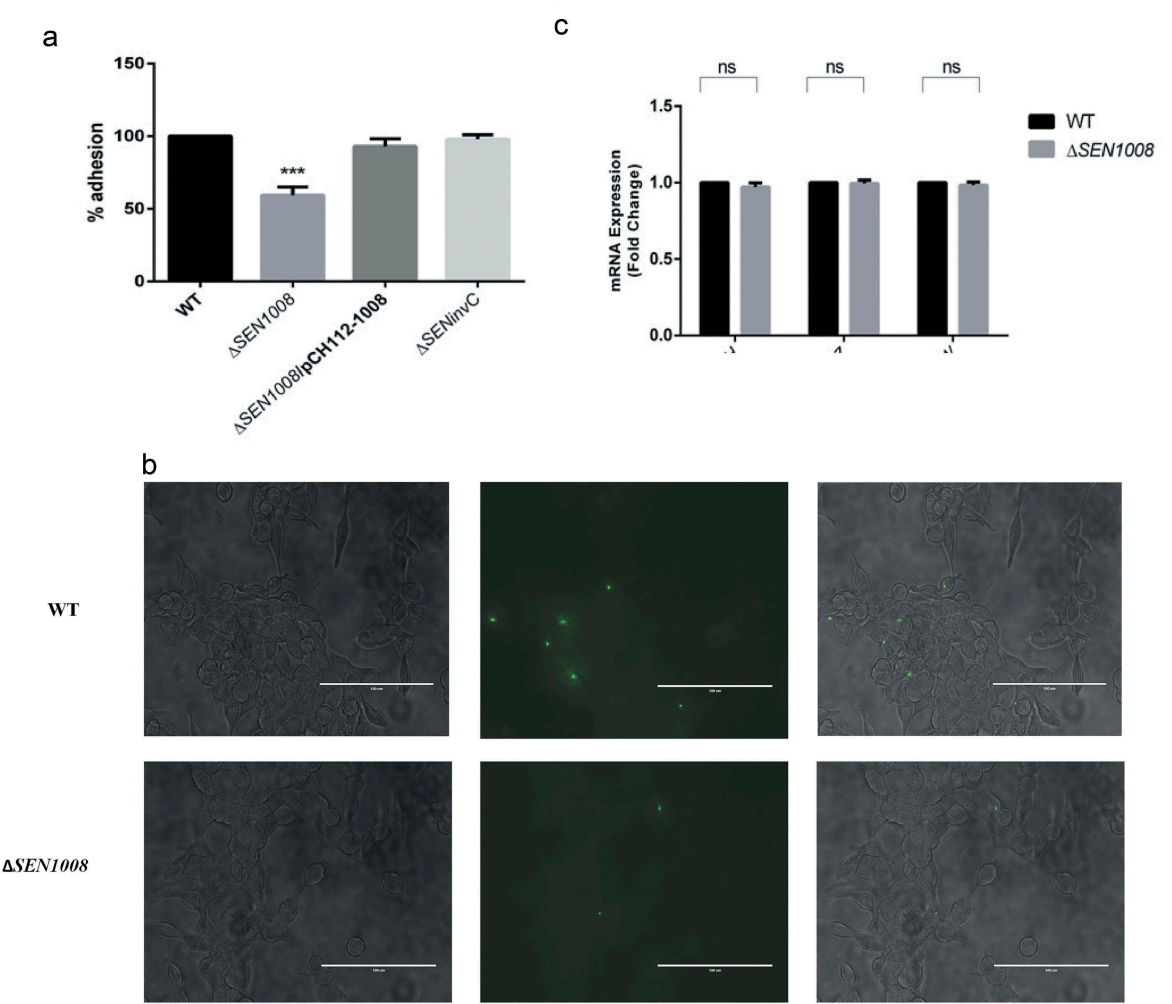
Bacterial Adhesion on colon cancer epithelial cells HCT116. (a) Adhesion assay with WT, Δ*SEN1008* and Δ*SEN1008*/pCH112-1008 in HCT116 showing the restoration of WT phenotype in the complemented strain. (b) Fluorescence microscopy to show adhesion of GFP-expressing WT and Δ*SEN1008* on HCT116 with a MOI of 20 bacteria/cell. *Salmonella* shown in green. Scale 100 µm. (c) Expression of genes encoding Type I fimbriae in WT*vs*Δ*SEN1008*throughqRT-PCR. The data were presented corresponding to the fold-change differences in gene expression in Δ*SEN1008*with respect to WT. Results were deduced from three independent experiments in triplicates and data represented as mean ± SD. ns, not significant (P > 0.05); Statistical significance: *P < 0.05, **P < 0.01, ***P < 0.001 (Student’s t-test).

The complemented strain (Δ*SEN1008*/pCH112-1008) was prepared and cell adhesion was checked to confirm the previously obtained adhesion phenotype [16]. HCT116 cells were infected with a MOI of 10 bacteria/cell,which showed 45% lesser adhesion for Δ*SEN1008* as compared to WT, whereas; the complemented strain restored the wild-type phenotype (Figure 1(a)). The same experiment, with a higher MOI of 20 bacteria/cell was repeated with GFP-expressing WT and mutant strains that displayed similar results 30-min post-infection in HCT116, when viewed under a fluorescence microscope (Figure 1(b)).

However, growth curve analysis in LB medium showed no difference between the mutant strain and the WT during 8 h of culturing (Fig. S1).

Now, adhesion of bacteria to host cells is usually affected by a lot of factors majorly involving type I fimbrial genes expression [4,10,29]. To further investigate this fact, qRT-PCR was done to check the mRNA expression of genes encoding type I fimbriae (Fim) in both wild-type and mutant. But, no significant difference in gene expression was observed for three different regulators of the operon such as FimZ, FimH, and FimW (Figure 1(c)).

### SEN1008 plays a role in bacterial motility and its uptake by macrophages

**Figure 2.**
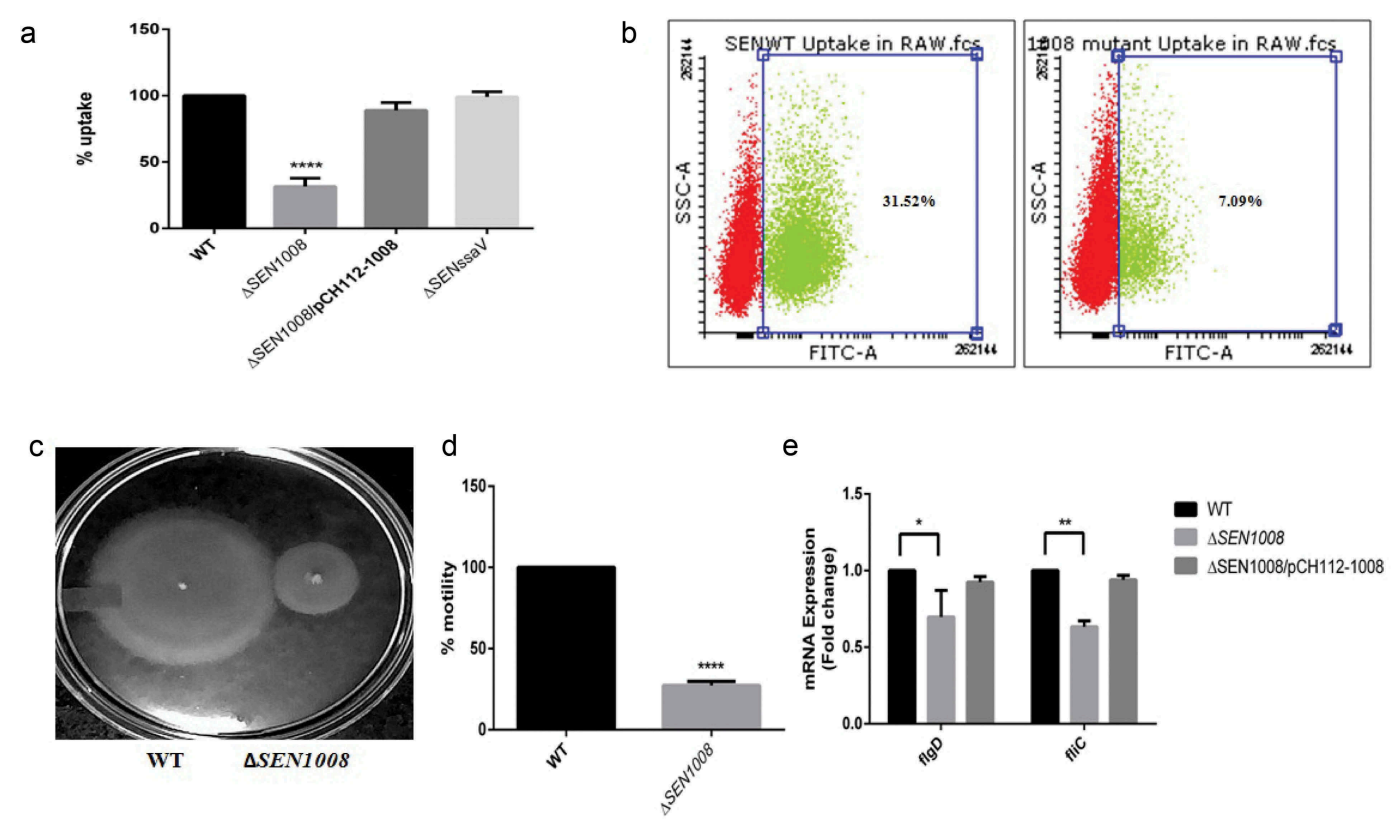
Bacterial uptake assay in murine macrophages RAW264.7 and analysis of motility. (a) Uptake of WT, Δ*SEN1008* and Δ*SEN1008*/pCH112-1008 by murine macrophages RAW264.7 at 2 h p.i. that shows restoration of WT phenotype in the complemented strain. (b) % uptake of GFP-expressing WT and Δ*SEN1008* by RAW264.7 at a MOI of 10 bacteria/cell after 2 h of infection by flow cytometry. (c) Motility assay was performed with WT and Δ*SEN1008* by placing 1 µl log-phase cultures of bacteria with same O.D on 0.3% LB agar plates. After 5 h of growth, the diameter of the growth region was measured. Scale in cm. (d) Bar diagram for % motility of *Salmonella*measured from diameters of the bacterial growth. (e) Expression of genes related to flagellarassemblyinWT*vs*Δ*SEN1008*throughqRT-PCR. Results were deduced from three independent experiments in triplicates and data represented as mean ± SD. ns, not significant (P > 0.05); Statistical significance: *P < 0.05, **P < 0.01, ***P < 0.001 (Student’s t-test).

After adhesion, the bacteria invade the host by breaching the intestinal epithelium and initiate the infection [30]. Upon entry, since, macrophages are important for bacterial engulfment and systemic spread [31,32], the bacterial uptake percentage by the macrophages was checked in murine macrophage cell line RAW264.7 with WT, mutant, and the complemented strain to confirm the earlier report. Only 30% of the mutant bacteria were sampled by the macrophages as compared to the wild-type and the complemented strain almost restored the original phenotype (Figure 2(a)). This was further confirmed by flow cytometry using GFP-expressing bacterial strains that showed 77% lesser uptake of Δ*SEN1008* as compared with wild-type by the macrophages (Figure 2(b)).

Now, the adhesion deficiency, as well as a huge difference in uptake by phagocytic cells triggered us to investigate the bacterial motility [33] in the mutant strain. As expected, there was more than 25% reduction in motility in case of Δ*SEN1008*(Figure 2(c,d).On the other hand,the complemented strain restored the wild-type phenotype (Fig. S2). To further investigate the loss of motility to be the probable reason behind a lesser uptake profile, the assay was re-performed with an additional step of slow centrifugation after infection to ensure proper contact between the bacteria and cell lines. This extra step increased the uptake process by a huge margin (Fig. S3), suggesting *SEN1008* to play significant roles in motility as well as macrophage-based uptake. Flagellar genes have always been attributed to be one of the major reasons behind reduced bacterial motility. In this regard, the flagellar assembly genes *fliC* and *flgD* were evaluated for their mRNA expressions in the strains. As expected, these genes were significantly downregulated in Δ*SEN1008* as compared with the WT and complemented strain (Figure 2(e)).

### SEN1008 helps in bacterial replication in macrophages and shows significant expression during ATR assay

**Figure 3.**
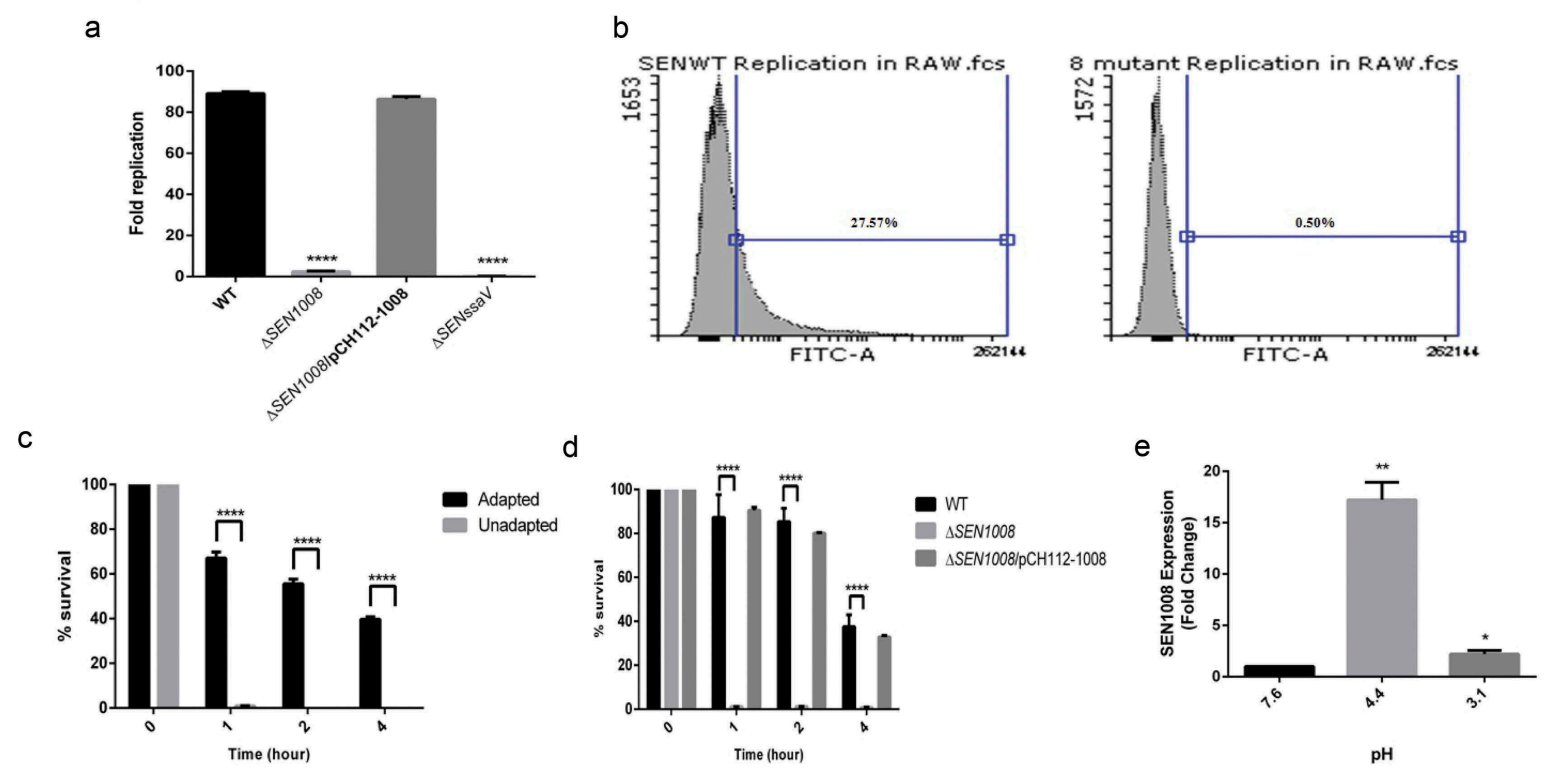
Bacterial intracellular replication assay in RAW264.7 murine macrophages and ATR assay. (a) Survival Assay of WT, Δ*SEN1008* and Δ*SEN1008*/pCH112-1008in RAW264.7 murine macrophages at 24 h time-point. (b) % survival of GFP-expressing WT and Δ*SEN1008* by RAW264.7 at a MOI of 10 bacteria/cell after 24 h of infection by flow cytometry. (c) The log-phase ATR of S. Enteritidis WT. (d) ATR assay of WT, Δ*SEN1008* and Δ*SEN1008*/pCH112-1008. (e)qRT-PCR analysis of *SEN1008* under normal (pH 7.6), adaptation (pH 4.4) and challenge (pH 3.1, 1 h) respectively. Results were deduced from three independent experiments in triplicates and data represented as mean ± SD. ns, not significant (P > 0.05); Statistical significance: *P < 0.05, **P < 0.01, ***P < 0.001 (Student’s t-test).

The ability of the bacteria to replicate itself in macrophages is a key virulent determinant during *Salmonella* infection [34]. To characterize this phenomenon, intracellular survival assay was done with the three strains in RAW murine macrophages. Δ*SENssaV*strain was used as a negative control for the study. Interestingly, Δ*SEN1008* was found to be highly attenuated in replication inside macrophages (Figure 3(a)). Ninety-eight percent of mutant bacteria were not able to survive at 24 hp.i. and this was again confirmed by repeating the experiment with WT and mutant bacterial strains transformed with GFP-expressing plasmids and analyzed by flow cytometer (Figure 3(b)).

Now, replication within SCVs in murine macrophages also depends upon the induction of ATR by the bacteria [14]. To study this phenomenon, wild-type strain was assessed for its role to induce effective ATR in MEM. The log-phase adapted cultures (grown in pH 4.4 for 1 h) and the log-phase un-adapted cultures (at pH 7.6) were exposed to extreme acid stress (pH 3.1) and bacterial viability was determined after 1, 2, and 4 h post-challenge. Obviously, the adapted cells could survive more than the un-adapted cells throughout the span of the experiment (Figure 3(c)). Next, to test the influence of *SEN1008* in this regard, ATR assay was performed with adapted cultures of WT, Δ*SEN1008* and Δ*SEN1008*/pCH112-1008 and it was found that unlike, the wild-type and complemented strain, the mutant could not mount an acid tolerance response and did not survive the challenge (Figure 3(d)). To negate the possibility of any growth deficiency, a growth kinetics study was performed in MEM which showed no significant difference between the three strains (Fig. S4). To further establish the role of *SEN1008*, during ATR, its mRNA expression was quantified in the wild-type strain after the adaptation phase and also 1 h after a challenge by qRT-PCR analysis. Interestingly, *SEN1008* expression was highly up-regulated (17 fold) post 1 h of adaptation (pH 4.4), while it came down to 2 fold post challenge (pH 3.1) (Figure 3(e)) with respect to its expression prior to acid stress (pH 7.6).

### ΔSEN1008 fails to cause systemic infection and acute colitis in mice at 3 days p.i.

**Figure 4.**
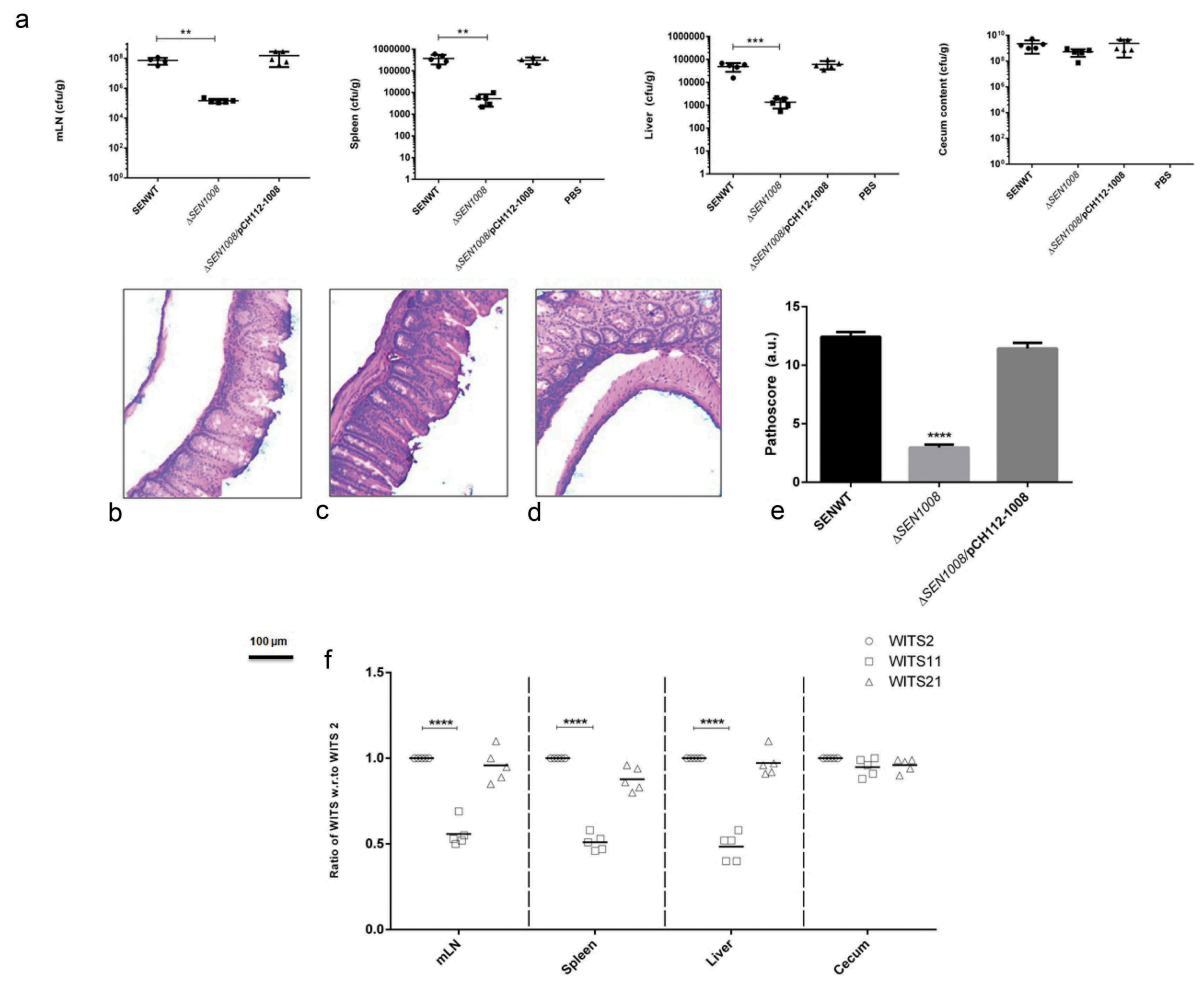
*In vivo* characterization of Δ*SEN1008*. Different groups of streptomycin-pretreated C57BL/6 mice were orally fed with ~10^7^cfu of WT, Δ*SEN1008*, Δ*SEN1008*/pCH112-1008 and PBS (negative control) separately. (a) Bacterial dissemination: At day 3 p.i., mice were euthanized and the bacterial load was enumerated in mLN, spleen, liver and cecum by plating on MacConkey agar. (b-d) Tissue histopathology: Hematoxylin-Eosin stained images for sections of mice cecal tissue (5 µm size) from WT, Δ*SEN1008* and Δ*SEN1008*/pCH112-1008 infected mice (left to right). Scale bar 100 µm. S, submucosal edema; Lp, lamina propria; L, lumen. (e) Determination of cecalpathoscore based on the parameters described in materials and methods.. (f) Assessment of competitive colonization by WITS- tagged WT (WITS2), Δ*SEN1008*(WITS11) and Δ*SEN1008*/pCH112-1008 (WITS21). Mice were infected with equal proportions of each strain with a total inoculum pool of ~10^7^cfu. At 72 h p.i., the mice were sacrificed and their mLN, spleen, liver, and cecum were collected and homogenized. The genomic DNA was isolated from each organ and the bacterial load of each strain was enumerated by qRT-PCR using WITS specific primers. The data were expressed as the ratio of WITS with respect to WITS21. Number of mice (n) = 5 for each group. Statistical significance: *P < 0.05, **P < 0.01, ***P < 0.001, ****P < 0.0001 (Student’s t-tests for Fig. 4A and 4E and Two-way ANOVA for Fig 4 F).

The *in vitro* virulence profile led us to do *in vivo* characterization of Δ*SEN1008*in a C57BL/6 streptomycin pre-treated mouse model. ~10^7^cfu of WT, mutant, and complemented strains were fed to each mice group (n = 5) by oral gavage. The control group was treated with PBS. At day 1 and day 2 p.i., the fecal shedding was estimated and found to be almost same for all the three groups indicating equivalent colonization in the gut (Fig S5A and S5B). At 3 days p.i., *Salmonella* was found to infect mLN, spleen and liver sufficiently in wild-type and complemented strain infected mice groups, whereas; the mutant-infected groups were systemically attenuated. Bacterial loads in cecum for all three groups were similar at 72-h p.i. (Figure 4(a)).

The cecalcryo-sections from all mice group were collected and HE stained for histopathological evaluation and as expected WT and complemented strain showed symptoms of acute inflammation while the mutant-infected group failed to cause sharp colitis by day 3 p.i (Figure 4(b-d)). Out of 13 arbitrary units of inflammation parameters, the path scores were observed to be 12 and 11 for WT and Δ*SEN1008*/pCH112-1008 infected tissues, while 2.8 for Δ*SEN1008* infected samples that corresponded to negligible to slight inflammation (Figure 4(e)).

Additionally, the bacterial loads in each tissue were also assessed through a competition-based mice infection assay where each mouse was orally administered with equal proportions of individually tagged WT, mutant and complemented strain (Fig. S6). The q-PCR results revealed significantly decreased colonization of systemic sites but almost similar cecal colonization by the mutant, in accordance with the previous *in vivo* findings (Figure 4(f)).

### SPI-2 effectors are down-regulated in ΔSEN1008 infected mice mln and spleen tissues

**Figure 5.**
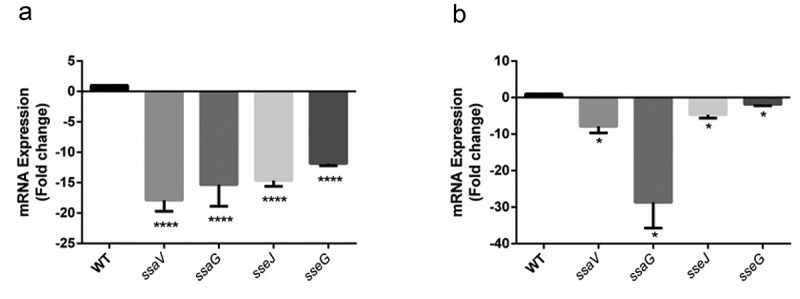
SPI-2 genes expression of WT vs. Δ*SEN1008* from infected mice tissues through qRT-PCR analysis. (a) Expression of SPI-2 genes in mLN; WT vs. Δ*SEN1008*.(b) Expression of SPI-2 genes in spleen; WT vs. Δ*SEN1008*. qRT-PCR was performed thrice in triplicate from each mice group (n = 5). Statistical significance: *P < 0.05, **P < 0.01, ***P < 0.001, ****P < 0.0001 (One way ANOVA).

Since, SPI-2 has been reported to be responsible for bacterial replication in macrophages and systemic colonization in mice [7,35]; the mRNA expressions of SPI-2 genes were quantified from mLN and spleen tissues of infected mice groups. The qRT-PCR analysis showed remarkable suppression of SPI-2 effectors (*sseJ*[14 fold in mLN; 4 fold in spleen], *sseG*[12 fold in mLN; 2 fold in spleen]) and genes encoding for secretory apparatus (*ssaV*[17 fold in mLN; 8 fold in spleen], *ssaG* [15 fold in mLN; 28 fold in spleen]) in mutant-infected mice as compared with that of WT (Figure 5(a,b). An additional qRT-PCR was also performed to determine the levels of SPI-2 effector proteins SseJ and SseG*in vitro* from overnight grown bacterial cultures, which showed 2.25 fold and 2.8 fold reductions, respectively, at the transcript level of the aforementioned genes in the mutant as compared to the WT (Fig. S7). However, in both *in vivo* and *in vitro*studies, the complemented strain had similar levels of SPI-2 effectors as that of WT (data not shown).

### Altered pro-inflammatory cytokine expression in serum of mice infected with WT and ΔSEN1008

**Figure 6.**
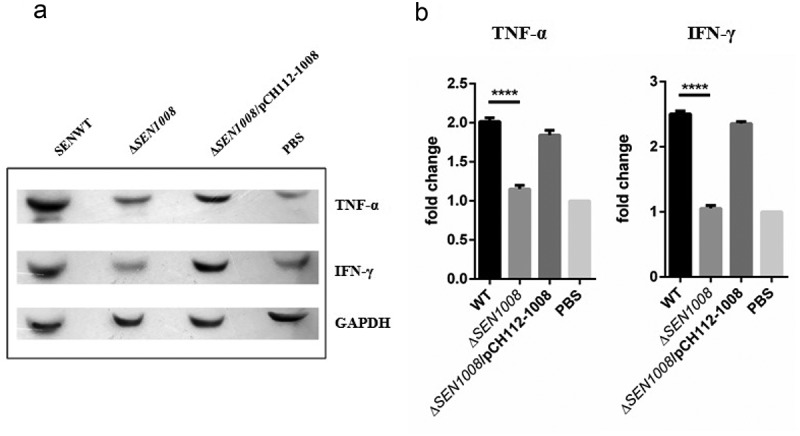
*In vivo* evaluation of pro- and anti–inflammatory cytokines from infected mice serum. (a) 50 µg of serum samples from indicated mice group were collected 72 h p.i. and run on 12% SDS PAGE; followed by transfer onto PVDF membrane, developed by western blot using appropriate primary (1:2000) and secondary (1:3000) antibodies in dark. (b) Quantification of the blots using ImageJ software. Statistical significance: *P < 0.05, **P < 0.01, ***P < 0.001, ****P < 0.0001 (Student’s t-test).

Cytokines are key players in the inflammation process [36]. Since, Δ*SEN1008*infected mice failed to show acute signs of inflammation; serum cytokine levels were observed through SDS PAGE, followed by subsequent western blot development and analysis using ImageJ. As expected, pro-inflammatory cytokines (TNF-α and IFN-γ) were significantly induced in WT and complemented strain than in the mutant and PBS treated mice (Figure 6(a,b)).

## Discussion

With gradual evolution, bacteria have also adapted themselves to fit into the ever-changing environment by modifying their own genome content and expression profiles of certain genes. The slightest variations in genome bring about phenotypic changes and host specificity even among different strains of the same species [37]. This phenomenon is usually observed in pathogenic bacteria leading to different symptoms and degrees of infection for a particular genus of bacteria [38]. Now, *Salmonella*, being a pathogen has undergone a lot of additions and deletions in its genome since its origin that has given rise to its different serovars and strains. Although, *S*. Enteritidis and *S*. Typhimurium share 98.98% of CDS, yet around 6.4% of genes are exclusively present in the former as compared to the latter [39]. This difference in genome content is assumed to have brought about changes in their degrees of pathogenesis and inflammation kinetics in mice model of infection [26]. However, the unique genes present in *S*. Enteritidis have not been studied in details and are still under investigation. One such island, ROD9 has been found to be present in *S*. Enteritidis but absent from *S*. Typhimurium and already a few genes have shown a role in virulence pathway of the same [40,41]. In this context, the present work focuses on another gene, *SEN1008* encoding for a putative exported hypothetical protein that showed certain sequence similarity with few other proteins such of *Legionella pneumophila*, another Gram-negative pathogen involved in gastroenteric diseases [42]. Those proteins are already reported for virulence associated functions in the latter such as EnhC [43] (enhanced entry protein), PilF [44] (fimbrial biogenesis and twitching motitlity protein) and also Type IV secretory system Dot/Icm proteins [45–47]. Moreover, in our previous study, Δ*SEN1008* strain had shown reduced adhesion on and uptake by epithelial and macrophage cell lines,respectively. All these findings led us to an in-depth analysis of the role of this gene with respect to *Salmonella* pathogenesis.

To find a potent role of *SEN1008*, a complemented strain (Δ*SEN1008*/pCH112-1008) was prepared and the adhesion and uptake assays were repeated confirming the observed phenotypes. Although there was no differential expression seen in type I fimbrial genes for the mutant strain as compared with the wild-type (Figure 1(c)), the adhesion was 40% lesser for Δ*SEN1008* (Figure 1(a)) indicating probable differences in expression of either other fimbrial genes or non-fimbrial adhesins. But, there was no significant variation found in the colonization of mice gut (Fig. S5) by the mutant strain as compared with the WT. However, motility was drastically affected upon deletion of *SEN1008* and this might have led to decreased adhesion and other *in vitro*phenotypes observed (Figure 2(a,b, S2) [48].However, the motility defect in the mutant strain was found to be due to suppressed expression of flagellar assembly genes in the same (Figure 2(e)). Besides this, the ability of the pathogen to replicate intracellularly in murine macrophage cell line was hugely attenuated for Δ*SEN1008*(Figure 3(a,b), suggesting its role in survival under stress conditions, as macrophages maintain a lower internal pH. Now, Type IV secretion proteins Dot/Icm of *L. pneumophila*have already been reported to be involved in bacterial replication in the hosts as well as in managing stress responses [46]. One such stress that *Salmonella* encounters in its course of infection are that of acid stress and the mechanism by which it survives a lethal pH is known as ATR [49]. In our work, we observed a remarkably higher expression of *SEN1008* under acid stress (Figure 3(e)) and the isogenic mutant strain failed to survive post-challenge (pH 3.1) even after the adaptation phase, pH 4.4 (Figure 3(d)). To this effect, we further characterized *SEN1008* for its possible role in virulence *in vivo.*

The *in vivo* mice experiment data revealed equal gut and cecal colonization for both the mutant and wild-type strains (Fig. S5 and 4(a)) but Δ*SEN1008* was found to be systemically attenuated. There was a significantly reduced bacterial density observed in mLN, spleen, and liver for mutant-infected mice group as compared to that of the wild-type and complemented strain-infected groups (Figure 4(a)). Additionally, there was no acute sign of cecal pathology identified in Δ*SEN1008* treated mice at 3 days p.i. (Figure 4(c,e)) as compared to WT and complemented challenged groups (Figure 4(b,d)). Since, there was significant levels of reduced systemic colonization (Figure 4(a)), decreased intracellular survival in macrophages (Figure 3(a)) by the mutant, it was necessary to elucidate the expression of genes functioning for SPI-2, as the latter has been extensively studied for its role in bacterial intracellular replication and systemic infection [50]. Serving as the major reservoirs of immune cells (such as macrophages), mLN and spleen tissues revealed a highly repressed expression of SPI-2 genes in Δ*SEN1008* infected mice when compared to that of the wild-type (Figure 5(a,b)), that correlated with the phenotypes observed earlier (Figures 3(a) and 4(a)). Also, SPI-2 is usually responsible for inducing late inflammation post 48 h of ingestion suggesting the probable reason for abated inflammation observed in case of mutant-infected cecal tissue (Figure 4(c)) [5]. Additionally, cecal pathology differences can also be explained by the production of cytokines, which play major roles in triggering inflammation [51,52]. A lot of factors such as cytokines, chemokines, etc. involving various signaling pathways leads to disintegrity of the epithelial membrane and are of great importance while studying host innate immune responses [36]. Clearly, there was a reduction in pro-inflammatory signals by the mutant strain (Figure 6(a,b) which was in accordance with the *in vivo* findings.

Together, this study reported a novel gene *SEN1008* to be immensely involved in acid stress survival of the pathogen and modulating SPI-2 genes expression, thereby participating in systemic colonization and cecal pathology in C57BL/6 mice model of *Salmonella* infection. Therefore, another hypothetical protein, SEN1008 from the ROD9 island, is identified as a potent virulence determinant in *S*. Enteritidis infection.
