# Supplementary material for: A ROD9 island encoded gene in Salmonella Enteritidis plays an important role in acid tolerance response and helps in systemic infection in mice
Source: Virulence. 2020 Mar 1;11(1):247–59. doi: 10.1080/21505594.2020.1733203 (PMC7051147; doi:10.1080/21505594.2020.1733203)
Supplement: Supplemental Material [file kvir-11-01-1733203-s001.doc]

**Supplementary information:**

**Table S1.** Primers used in the study

| **Primer** | **Sequence (5’-3’)** | **Description** | **Reference** |
| --- | --- | --- | --- |
| Fw 16srRNA | TTCCAGTGTGGCTGGTCATC | Housekeeping genes for *Salmonella* | [16] |
| Rw 16srRNA | TGCCTGATGGAGGGGGATAA |
| Fw gmk | AGCAAATTCGCGAAAAGATG |
| Rw gmk | TGGCAATGACTTCTTCGCTAT |
| WITS-11 | ATCCCACACACTCGATCTCA | Confirmatory primers for WITS tagging | **[26]** |
| WITS-2 | ACCCGCAATACCAACAACTC |
| WITS-21 | ACAACCACCGATCACTCTCC |
| ydgA-Fw | GGCTGTCCGCAATGGGTC |
| Fw pCH 1008NcoI | AATTATCCATGGTATACACCAATAACAAGGGGGGGA | Cloning primers for  SEN1008 complementation | This study |
| Rw pCH 1008XbaI | GCGCGGTCTAGATTATAAATAATTTTCTGGCTTG |
| Fw 1008 | AGCAGAACAGGGGGATATGAAAG | Real-time primers for SEN1008 expression | This study |
| Rw 1008 | CCCCCTTTGCCATCCATGAA |
| Fw sseJ | CCGGAAGCTTTTGGTCTTGC | SPI-2 secretion apparatus and effectors | [21] |
| Rw sseJ | GCCGATGTACTTCCCCCTTC |
| Fw sseG | GGTCATTGCCATCCCATCCA |
| Rw sseG | TTGCCTATGGCTCACGACAG |
| Fw ssaV | GTGGATGCTTTCGGTAAGTTTGTC |
| Rw ssaV | CGATACTCATTTGTTTGCCTGGC |
| Fw ssaG | TTAGTGGATATGCTCTCCCACA |
| Rw ssaG | CGCTTTAATCATCGATTCTGGGT |
| Fw FimZ | CTTTTGCGGCGATTTGCACT | Type I fimbrial genes | This study |
| Rw FimZ | TGCAGCAGATCGTGGGAAAT |
| Fw FimH | GTTTATGTCATGACCGCCGC |
| Rw FimH | TTGACCTGCGAATCCAGACC |
| Fw FimW | TGGCCCTGGATGATGATTGG |
| Rw FimW | ATGCCAGAAGGGACGCTATG |
| Fw FliC | TCGCGTAGTCGGAATCTTCG | Flagellar assembly genes | [16] |
| Rw FliC | CTTTGGCACAGGTTGACACG |
| Fw FlgD | TTGGCGTGGAATTGCAACAG |
| Rw FlgD | TACCGTTGTTCCGTCCGTTT |

**
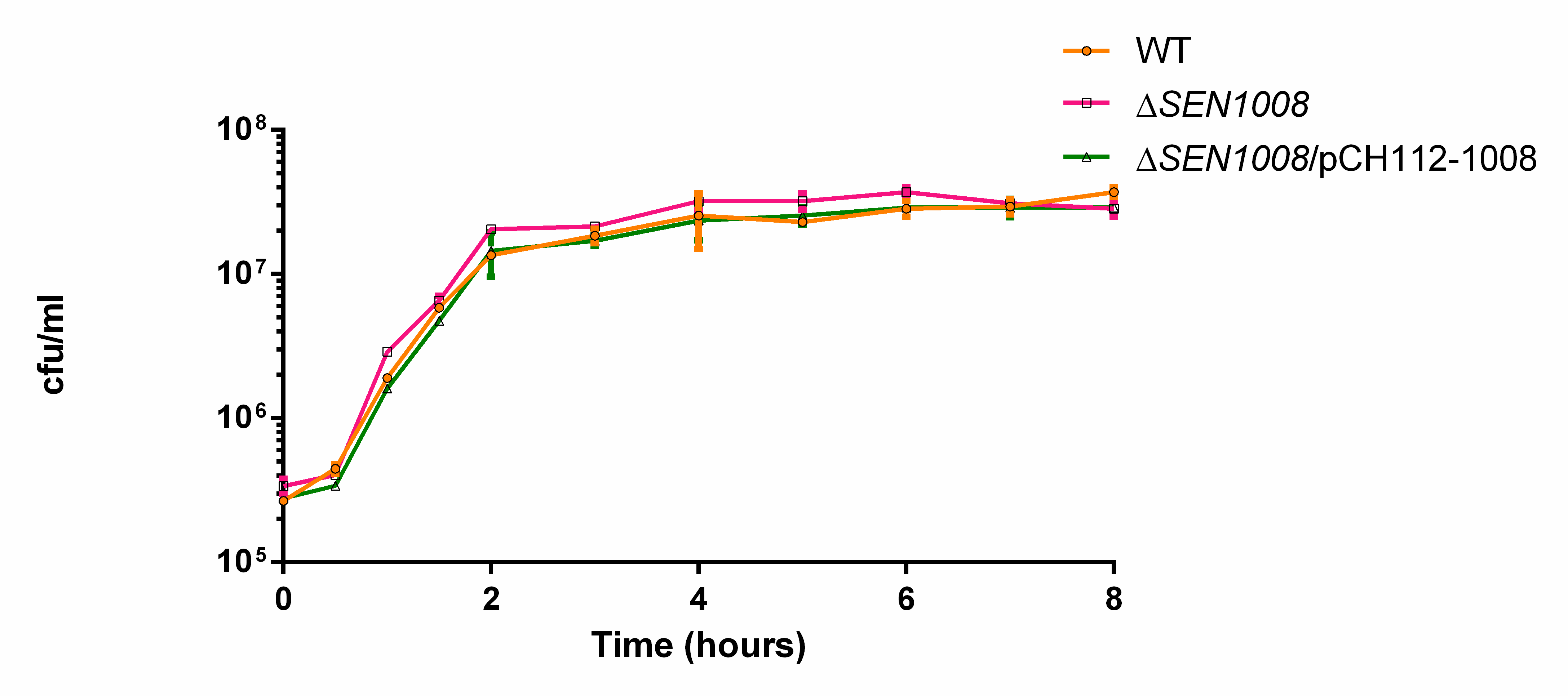
**

**Figure S1.** Growth curve experiment to check the growth pattern in WT, Δ*SEN1008* and Δ*SEN1008*/pCH112-1008 in LB media in the form of cfu counting at different time intervals till 8 hours. The experiment was performed thrice in triplicates and data represented as mean ± SD.


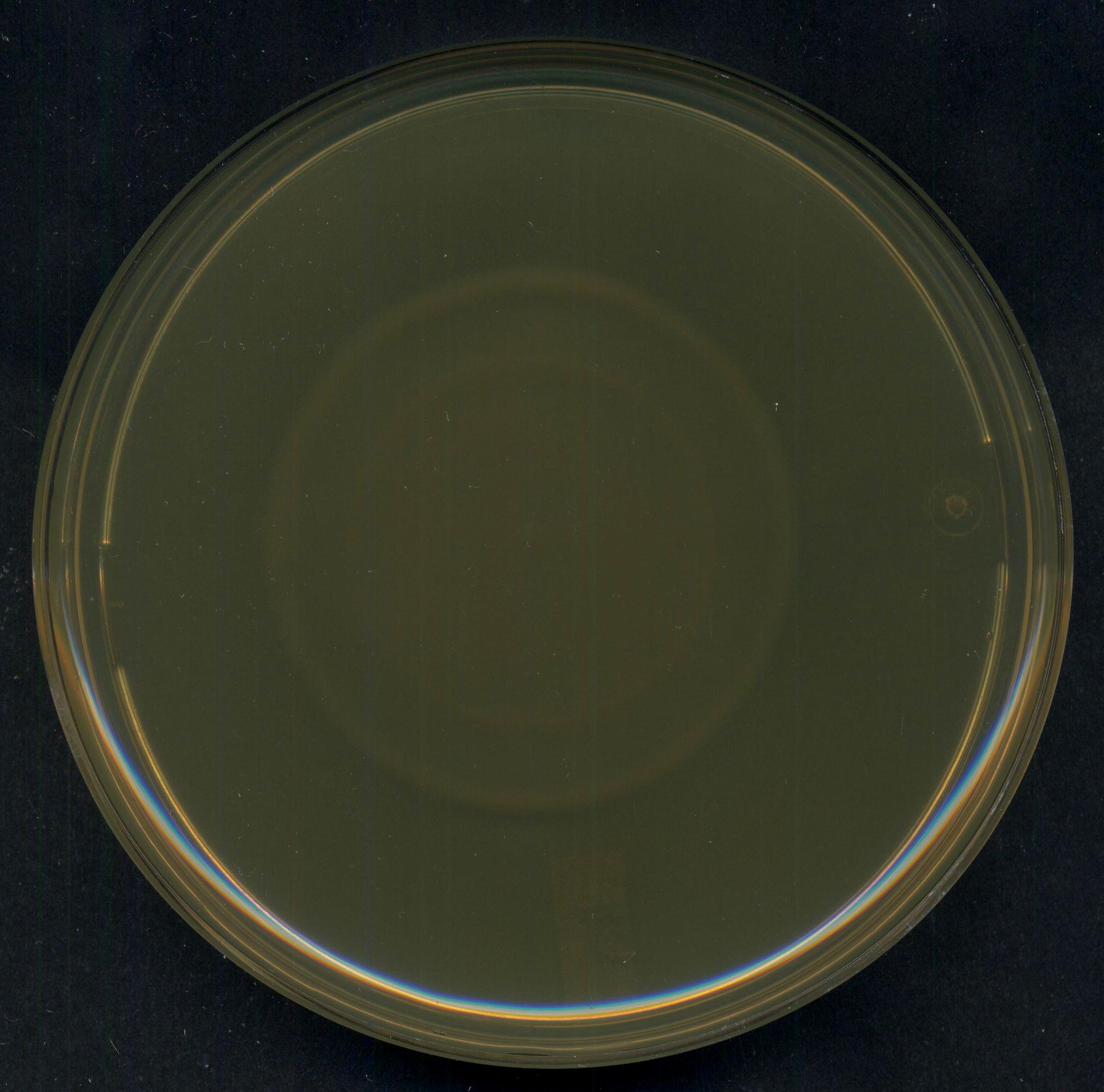

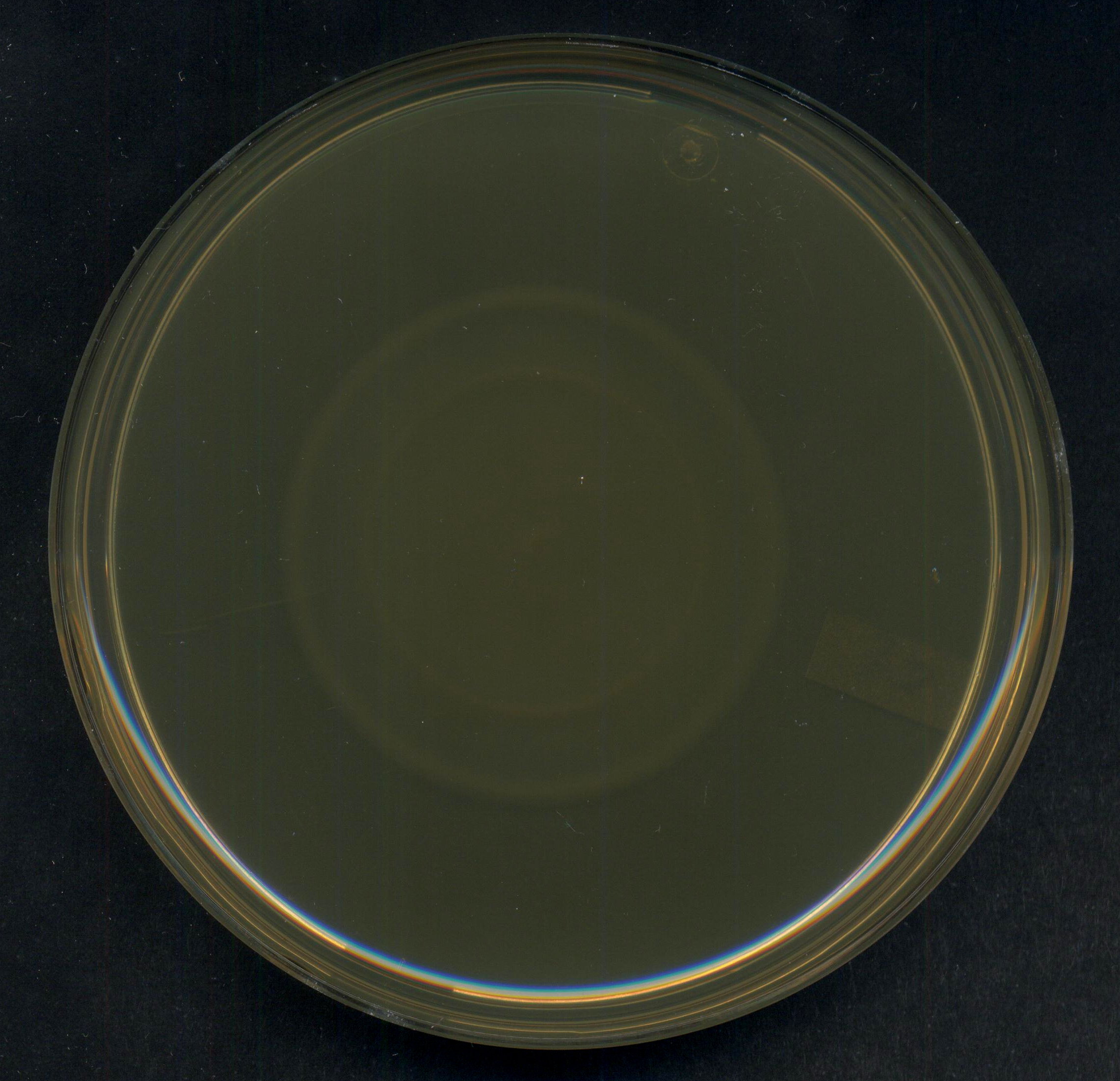

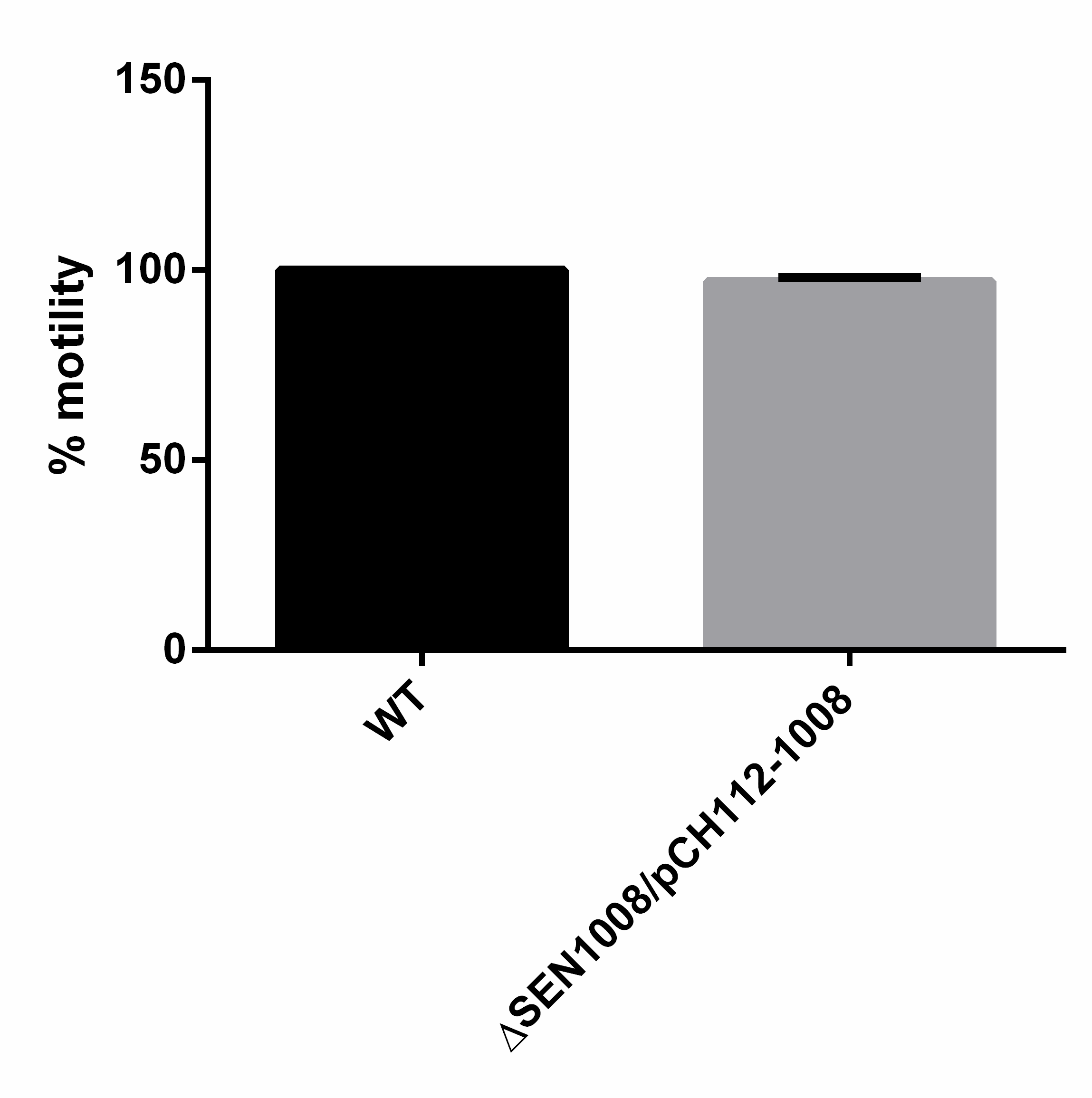


(A) (B) (C)

**Figure S2.** Motility assay was performed with (A) WT and (B) Δ*SEN1008*/pCH112-1008 by placing 1µl log phase cultures of bacteria of same O.D on 0.3% LB agar plates. After 5h of growth, the diameter of growth region was measured. Scale in cm. (C) Bar diagram showing % motility of *Salmonella* measured from diameters of the bacterial growth. Results were deduced from three independent experiments in triplicates and data represented as mean ± SD. ns, not significant (P>0.05); Statistical significance: *P < 0.05, **P < 0.01, ***P < 0.001 (Student’s t-test)


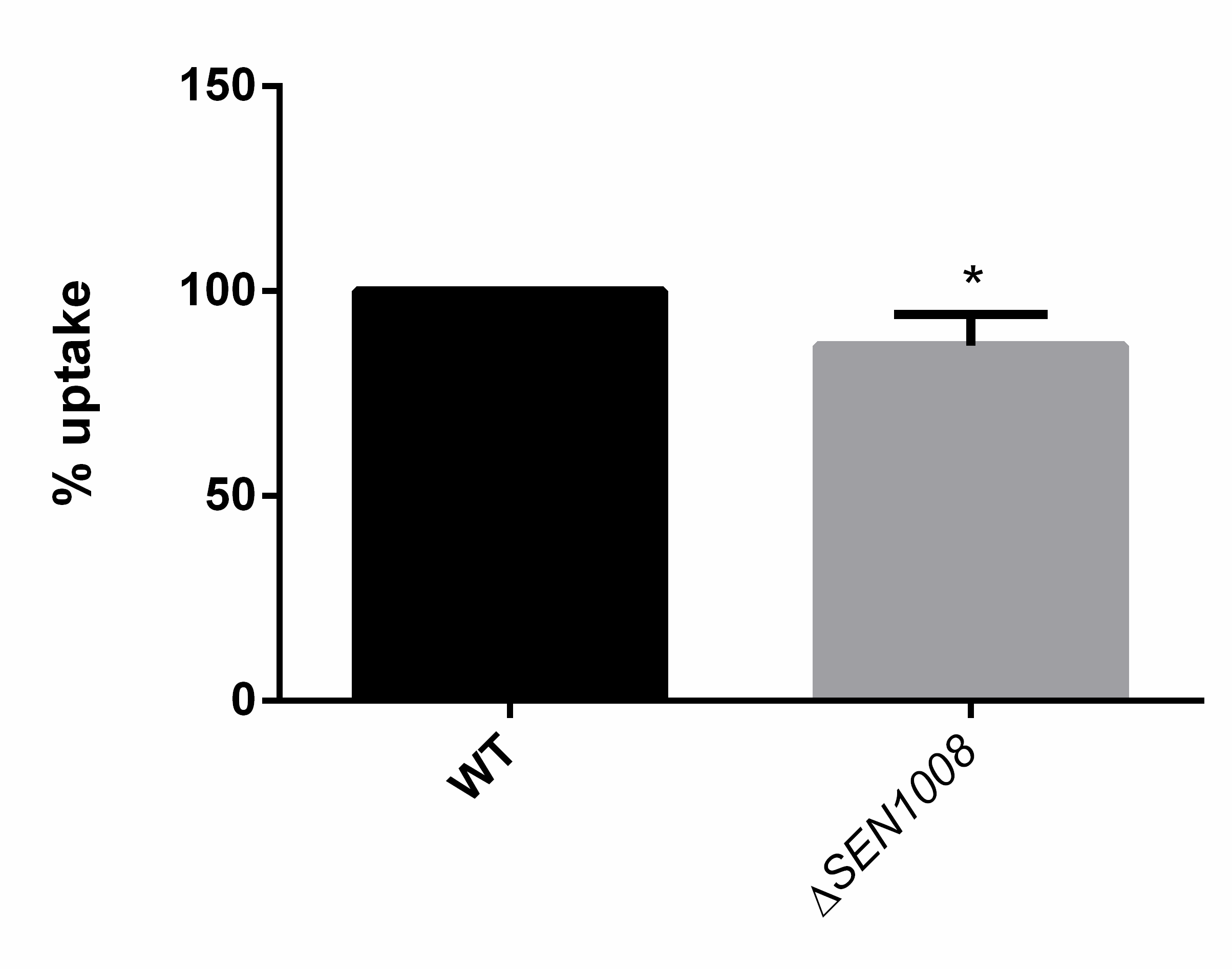


**Figure S3.** Uptake assay of WT, Δ*SEN1008* by murine macrophages RAW264.7 with an added centrifugation step at 500xg for 5 minutes immediately after infection to ensure proper contact between bacteria and host cells. The experiment was performed thrice in triplicates and data represented as mean ± SD (Student’s t-test).


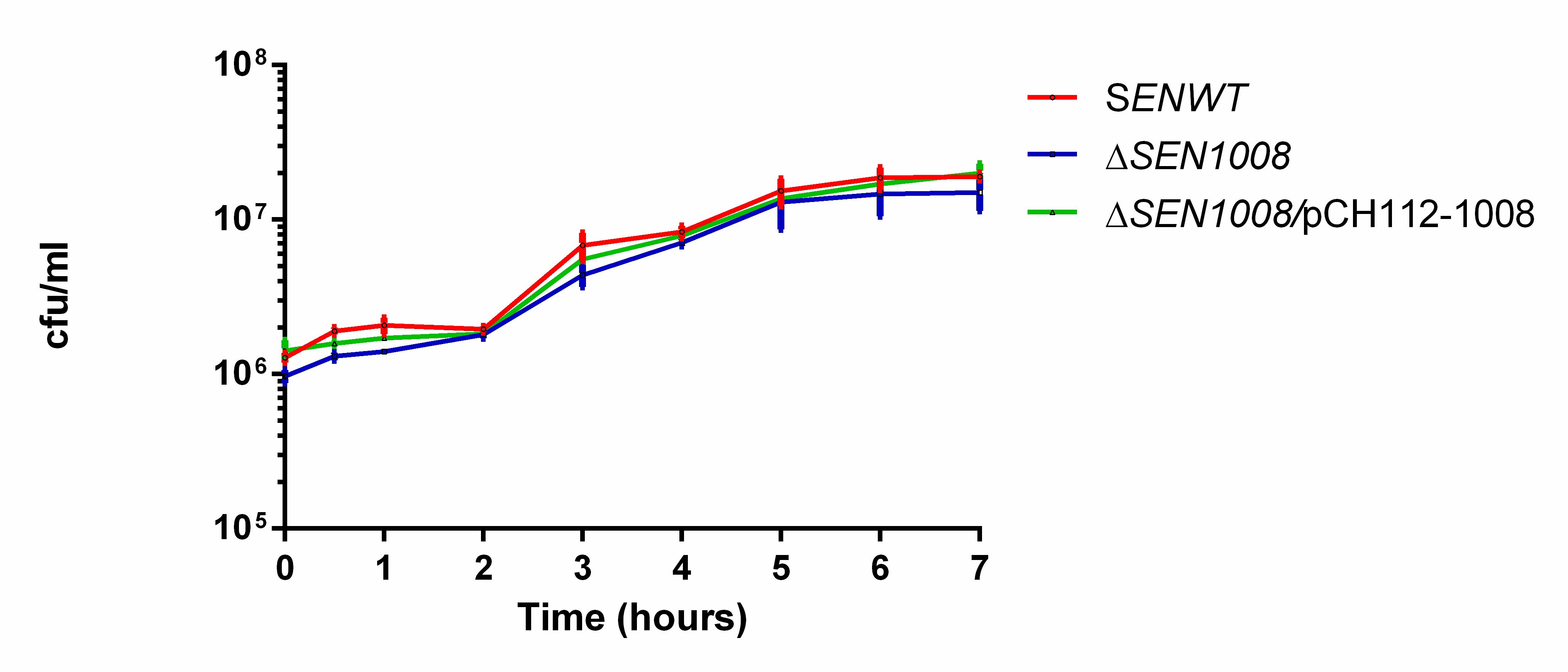


**Figure S4.** Growth curve experiment to check the growth pattern in WT, Δ*SEN1008* and Δ*SEN1008*/pCH112-1008 in MEM in the form of cfu counting at different time intervals till 7 hours. The experiment was performed thrice in triplicates and data represented as mean ± SD.


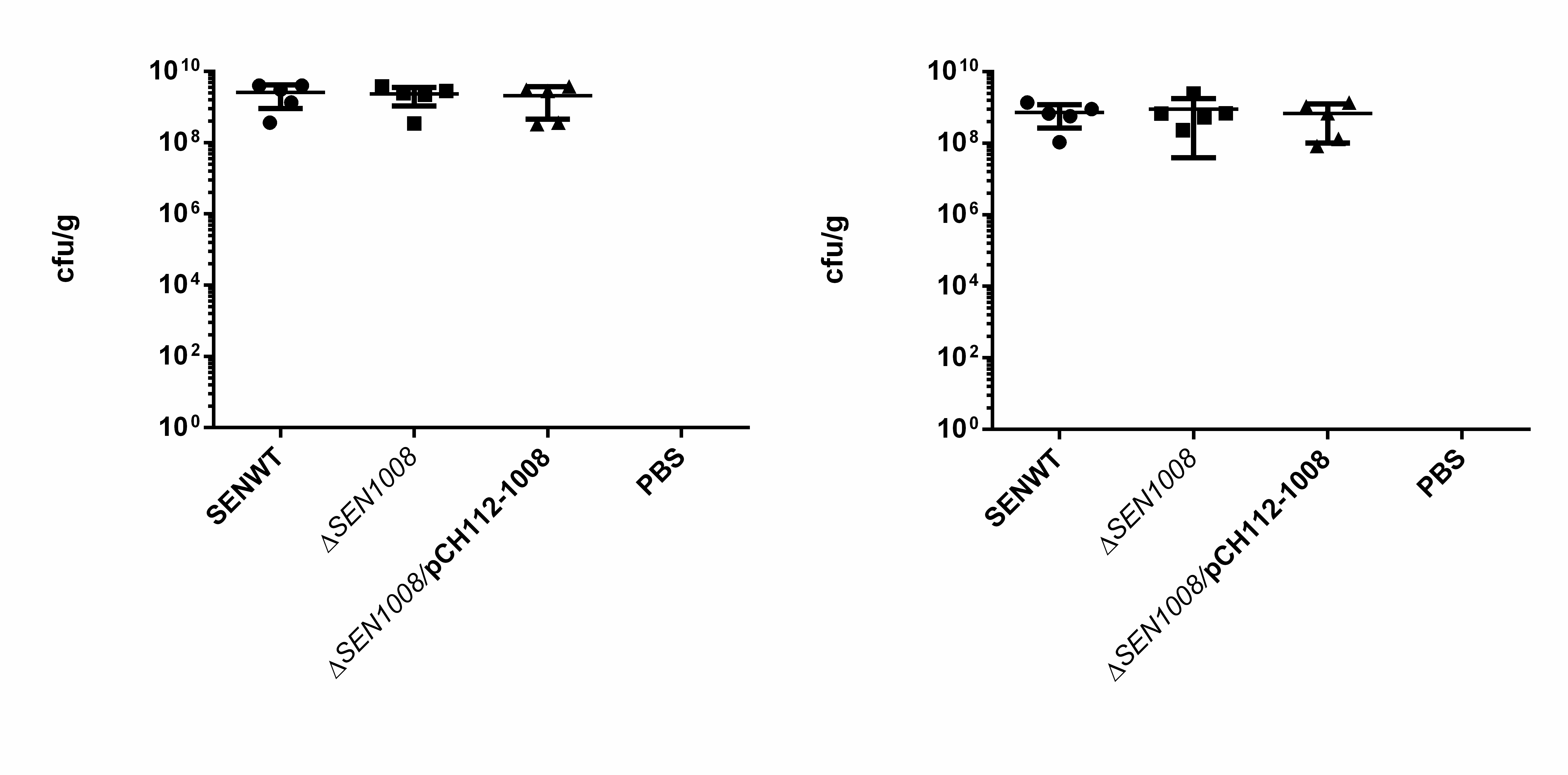


**Figure S5.** Fecal shedding of different *Salmonella* strains from streptomycin-pretreated C57BL/6 mice (n=5) orally fed with ~10^7^ cfu of WT, Δ*SEN1008*, Δ*SEN1008*/pCH112-1008 and PBS (negative control) separately as analyzed by plating at day 1 and day 2 p.i.


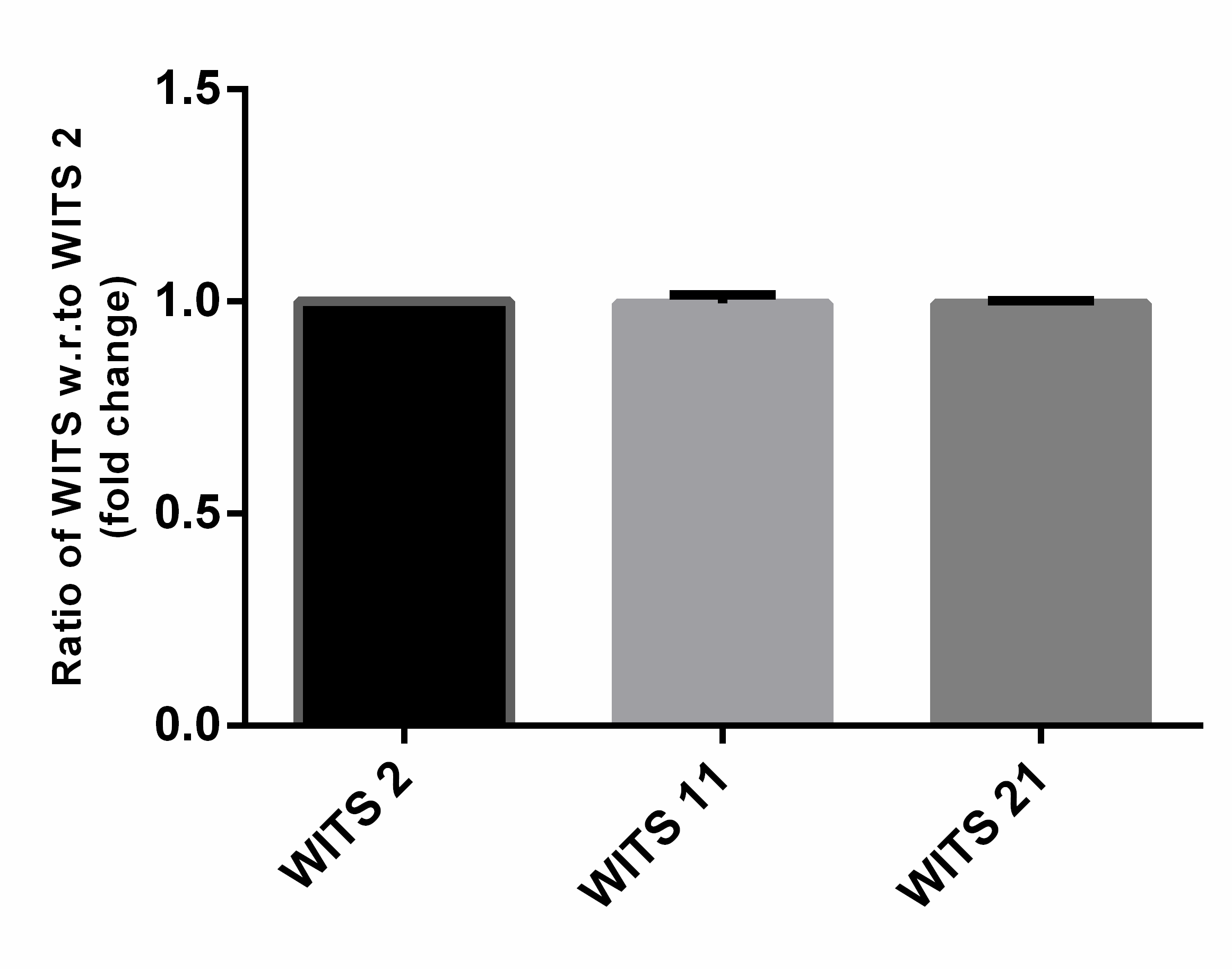


**Figure S6.** Assessment of pre-inoculum density by qPCR. *S*. Enteritidis was tagged with WITS as follows: WT (WITS 21), Δ*SEN1005* (WITS 2) and Δ*SEN1005*/pCH112-1005 (WITS 1). Genomic DNA was isolated from the mix inoculum pool and proportion of each strain was obtained by qPCR with WITS specific primers. The data was represented as ratio of WITS 2 and WITS 1 with respect to WITS 21 (WT) present in the inoculum.


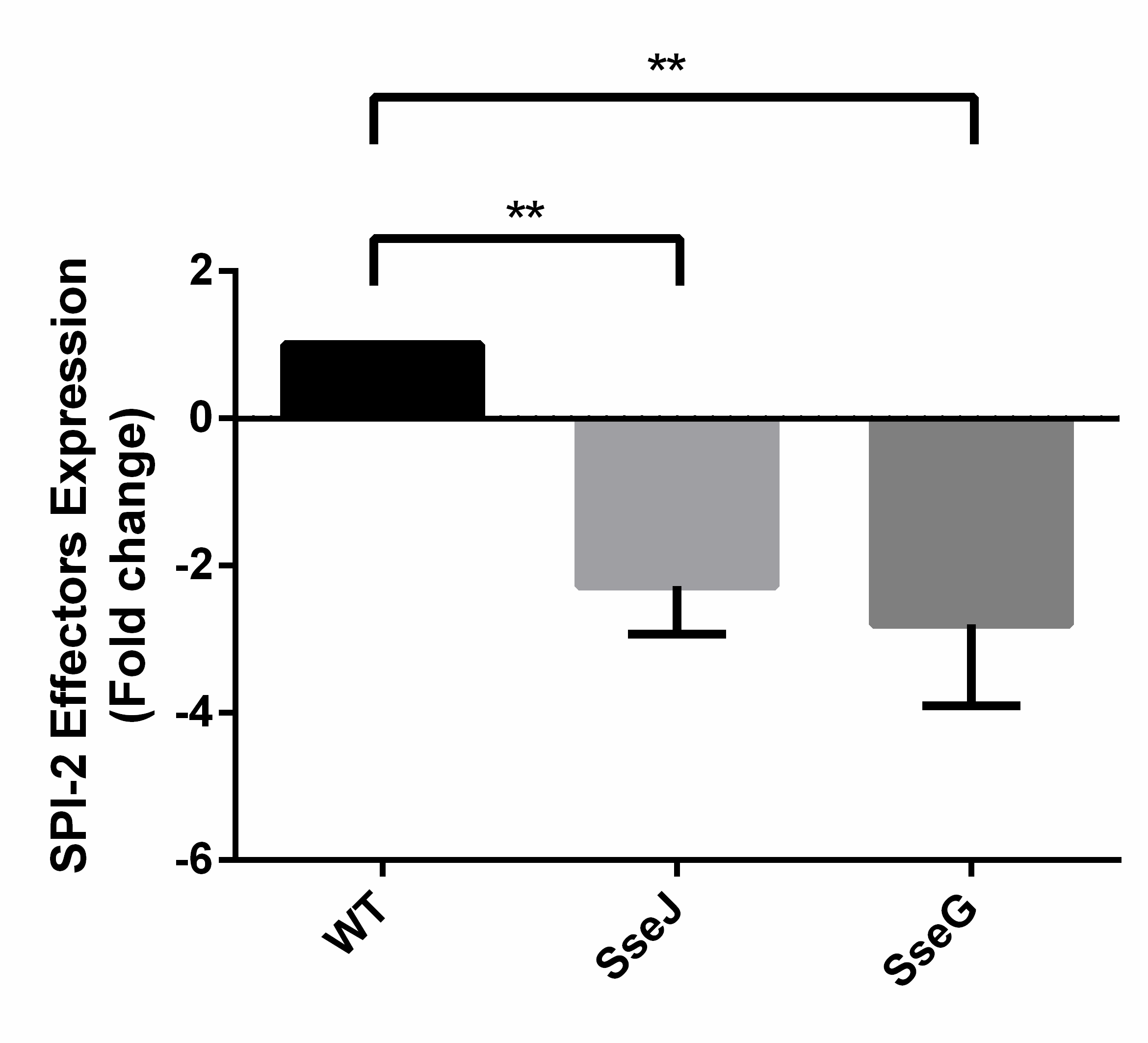


**Figure S7.** SPI-2 effector genes expression from overnight grown bacterial cultures of WT vs. Δ*SEN1008* through qRT-PCR analysis. The experiment was performed thrice in triplicate. Statistical significance: *P < 0.05, **P < 0.01, ***P < 0.001, ****P < 0.0001 (One way ANOVA).
